# Arterial blood sampling in male CD-1 and C57BL/6J mice with 1% isoflurane is similar to awake mice

**DOI:** 10.1152/japplphysiol.00640.2018

**Published:** 2018-10-04

**Authors:** Ashley M. Loeven, Candace N. Receno, Caitlin M. Cunningham, Lara R. DeRuisseau

**Affiliations:** ^1^Department of Biological and Environmental Sciences, Le Moyne College, Syracuse, New York; ^2^Department of Mathematics, Statistics and Computer Science, Le Moyne College, Syracuse, New York

**Keywords:** Paco_2_, pH, pulse oximetry

## Abstract

Isoflurane (ISO) is a commonly used anesthetic that offers rapid recovery for laboratory animal research. Initial studies indicated no difference in arterial Pco_2_ ($Pa_{CO}{}_{{}_{2}}$) or pH between conscious (NO ISO) and 1% ISO-exposed CD-1 mice. Our laboratory investigated whether arterial blood sampling with 1% ISO is a suitable alternative to NO ISO sampling for monitoring ventilation in a commonly studied mouse strain. We hypothesized similar blood chemistry, breathing patterns, and cardiovascular responses with NO ISO and 1% ISO. C57BL/6J mice underwent unrestrained barometric plethysmography to quantify the pattern of breathing. Mice exposed to hypoxic and hypercapnic gas under 1% ISO displayed blunted responses; with air, there were no breathing differences. Blood pressure and heart rate were not different between NO ISO and 1% ISO-exposed mice breathing air. Oxygen saturation was not different between groups receiving 2% ISO, 1% ISO, or air. Breathing frequency stabilized at ~11 min of 1% ISO following 2% ISO exposure, suggesting that 11 min is the optimal time for a sample in C57BL/6J mice. Blood samples at 1% ISO and NO ISO revealed no differences in blood pH and $Pa_{CO}{}_{{}_{2}}$ in C57BL/6J mice. Overall, this method reveals similar arterial blood sampling values in awake and 1% ISO CD-1 and C57BL/6J mice exposed to air. Although this protocol may be appropriate in other mouse strains when a conscious sample is not feasible, caution is warranted first to identify breathing frequency responses at 1% ISO to tailor the protocol.

**NEW & NOTEWORTHY** Conscious arterial blood sampling is influenced by extraneous factors and is a challenging method due to the small size of mice. Through a series of experiments, we show that arterial blood sampling with 1% isoflurane (ISO) is an alternative to awake sampling in C57BL/6J and CD-1 male mice breathing air. Monitoring breathing frequency during 1% ISO is important to the protocol and should be closely followed to confirm adequate recovery after the catheter implantation.

## INTRODUCTION

Conscious arterial blood sampling is the gold standard to confirm ventilation changes in humans and animals. Cannulation for this technique has its challenges, especially in small laboratory animals such as mice, due to size and catheter patency (3, 5). There are elegant studies that use repeated blood sampling with high success in mice (20, 24), whereas others have demonstrated elevated markers of stress due to handling and sample collection procedures innate to the technique (reviewed in Ref. 4). With the increased use of mice in respiratory research, uncovering a more feasible way to monitor arterial blood would complement breathing studies.

Other methods of arterial blood collection include cardiac puncture in mice (7, 37, 39), and following decapitation (6). Although these techniques are suitable for terminal experiments, we were interested in a protocol for chronic studies of ventilation that would be representative of quiet breathing in mice. The cannulation model explored in this paper is the only currently available method for obtaining arterial blood samples in vivo. Several measures are available for venous sampling (2a), but to monitor ventilation it is important to sample from the arterial system (reviewed in Ref. 8). Results from this investigation are applicable not only to respiratory physiology experiments, but also to other studies that use a nonsurgical plane of anesthesia, such as noninvasive imaging and ultrasound (29, 32).

We explored a method to quantify arterial Pco_2_ ($Pa_{CO}{}_{{}_{2}}$) and pH in mice anesthetized with 1% isoflurane (ISO) and compared it with conscious (NO ISO) measures in two common strains of mice: CD-1 and C57BL/6J. The purpose was to reduce extraneous factors faced when quantifying conscious sampling (hyperventilation, excitement of the mouse, feasibility of connecting the tether, movement, et cetera; Refs. 3, 5, 23, 34), but it was unknown whether this level of ISO would influence ventilation. A previous study by Massey et al. (27) demonstrated similar minute ventilation and tidal volume in mice with 1% ISO and NO ISO, suggesting that this amount of anesthesia does not alter pattern of breathing in room air conditions.

To inform ventilation measures, we also considered heart rate and blood pressure responses in NO ISO and 1% ISO mice. Previous cardiovascular ISO reports have focused on the response to various anesthetics with longer-term use (11, 35), making fewer comparisons with awake mice. However, one group found a 20% depression in mean arterial pressure with 1.5–2% ISO compared with baseline for several mouse strains (21). This same report also found declines in heart rate, which was in line with two other studies (36, 42) comparing 1.5–2% ISO with awake mice. In all cases, decreases in cardiovascular function were to a smaller degree than with other anesthetics. The study by Tsukamoto et al. (36) reporting lower heart rate with ISO also found a decrease in oxygen saturation after 5% ISO anesthesia was initiated, where values hovered ~95% with continued (2%) administration. This decline in oxygen saturation is of interest to note, as it coincided with a depression in breathing frequency. Since a higher concentration is used in the previous literature, the exact cardiorespiratory response at 1% ISO is unknown. In addition to arterial blood sampling as the final measure of ventilation, the utilization of blood pressure and pulse oximetry allows for a more integrative approach when considering the 1% ISO vs. conscious model.

This investigation aimed to test the hypothesis that 1% ISO-anesthetized animals would display a similar ventilatory phenotype as NO ISO mice. Arterial blood samples were collected during the 5th min of 1% ISO exposure in CD-1 and C57BL/6J mice. We continued investigating cardiorespiratory responses in C57BL/6J mice administered NO ISO and 1% ISO. The pattern of breathing was quantified in C57BL/6J mice exposed to air, hypoxic gas, and hypercapnic gas. These experiments were performed to confirm previous reports that NO ISO and 1% ISO responses are not different when breathing room air, but 1% ISO does blunt the ability to respond to hypercapnia compared with NO ISO (27, 28). The addition of hypoxic gas allowed for characterizing another respiratory challenge with NO ISO and 1% ISO. Further experiments were conducted in which blood pressure and heart rate were measured to quantify any changes in the cardiovascular system with this level of anesthesia, as any alterations in these variables could influence gas exchange (15). Pulse oximetry was implemented to monitor the minute-to-minute oxygen saturation levels during NO ISO and 1% ISO. Arterial blood sampling, breathing patterns, blood pressure, and oxygen saturation were quantified to evaluate whether physiological differences emerged in NO ISO and 1% ISO mice.

## MATERIALS AND METHODS

### Animals

C57BL/6J (*n* = 24; blood sampling, blood pressure, pulse oximetry, barometric plethysmography, piezoelectric breathing frequency experiments) and CD-1 (*n* = 10; blood sampling experiments) 2-mo-old male mice were delivered from The Jackson Laboratory (Bar Harbor, ME). Mice were kept on a 12:12-h light-dark cycle, group-housed, and fed ad libitum with standard rodent chow (Teklad 22/15 Rodent Diet 8640; Envigo, Huntingdon, United Kingdom) and constant access to water. Physiological experiments were performed on mice aged 3–5 mo. All procedures were submitted to and approved by the Le Moyne College Institutional Animal Care and Use Committee.

### Experimental Design

**Fig. 1.**
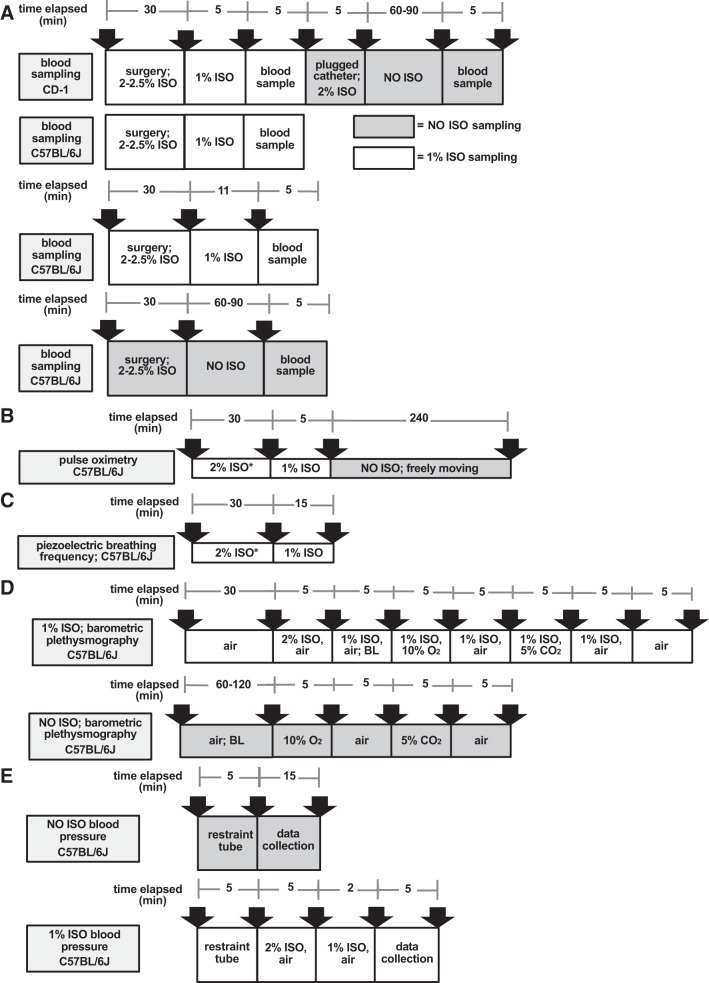
Timeline figure for all protocols. *A*: arterial blood sampling was conducted in both CD-1 and C57BL/6J male mice under conscious (NO ISO) and 1% isoflurane (1% ISO) conditions in air. Data are presented in Table 1. *B*: pulse oximetry used to monitor oxygen saturation when the mouse was on the surgical table [2% isoflurane (2% ISO) and 1% ISO] followed by recovery (NO ISO) in air. This measure was to confirm that the NO ISO sample was taken at an appropriate point in recovery. *First 3 min were with 3% isoflurane (3% ISO) for induction of anesthesia; 2% ISO reported value was averaged for the last 5 min of exposure. Data are presented in Fig. 2. *C*: piezoelectric breathing frequency collected on the surgical table to simulate the ISO blood sampling setup. This experiment was used to quantify breathing frequency during 2 and 1% ISO. *First 3 min were with 3% ISO for induction of anesthesia; 2% ISO reported value was averaged for the last 5 min of exposure. Data are presented in Fig. 3. *D*: breathing frequency, tidal volume, minute ventilation, and tidal volume/inspiratory time collected with barometric plethysmography. These experiments were a proof of concept to confirm no changes in the pattern of breathing with NO ISO vs. 1% ISO in air; additional exposures of hypoxia and hypercapnia were administered to identify possible changes in the pattern of breathing in response to NO ISO and 1% ISO. BL, baseline collection. Data are presented in Figs. 4 and 5. *E*: blood pressure data were collected using a volume-pressure recording tail cuff with NO ISO and 1% ISO. The purpose of this experiment was to identify possible changes in cardiovascular function with NO ISO vs. 1% ISO. Data are presented in Table 2.

The primary goal of these investigations was to determine whether awake and 1% ISO-administered mice had similar ventilation, suggesting that an arterial blood sample is representative of conscious air breathing at 1% ISO. To test this overarching research question, CD-1 and C57BL/6J mice had arterial blood samples immediately following implantation of a catheter. All initial studies were done using a 5-min 1% ISO exposure (timeline in Fig. 1*A*) following ~30 min at 2–3% ISO. Although the experiments were not simultaneous, this timeline was followed for both arterial blood sampling and pulse oximetry (timeline in Fig. 1, *A* and *B*). Once ISO was stopped, NO ISO responses were followed for 60–90 min during arterial blood sampling experiments and 4 h in the case of pulse oximetry. Initial results indicated a lower pH (although a nonacidic value) for C57BL/6J mice using the 5-min 1% ISO timeframe vs. NO ISO [NO ISO: 7.43 (0.02), 1% ISO: 7.38 (0.03); *P* = 0.007]. Additional studies were completed to determine an optimal time for a 1% ISO C57BL/6J arterial sample. These experiments used the same 30 min of 2–3% ISO, followed by 15 min of 1% ISO for observation of breathing frequency on the surgical table (timeline in Fig. 1*C*). An 11-min recovery at 1% ISO was observed as appropriate for C57BL/6J mice; arterial blood samples were then collected in an experiment with ~30 min of 2–3% ISO followed by 11 min of 1% ISO. Repeatability and proof-of-concept studies were completed to monitor the pattern of breathing and blood pressure in NO ISO and 1% ISO-exposed mice. These experiments used 2% ISO for 5 min followed by 1% ISO exposure (timeline in Fig. 1, *D* and *E*).

### Arterial Blood Sampling

C57BL/6J and CD-1 male mice were used in the blood sampling experiments. Anesthesia induction was performed with 3% ISO at a flow rate of 500–800 ml/min followed by anesthetic maintenance using a nose cone with 2–2.5% ISO at the same flow rates. The nose cone had a vacuum flow tube set at ~100 ml/min to scavenge excess ISO. Before beginning the surgery, depth of anesthesia was confirmed and eye protectant administered. A far-infrared heating platform (Kent Scientific, Torrington, CT) was used throughout the procedure for animal warming. Fur was removed and an alcohol and betadine scrub used to clean the surgical site. An incision was made and tissue reflected to view the femoral artery. The artery was isolated and ligated distally, whereas the proximal end was clamped with a Roboz 2A butterfly clamp (RS-6472; Gaithersburg, MD). A 30-gauge needle bent at 90° punctured the artery, and the catheter was placed into the artery and tied down. Following removal of the clamp, the catheter was advanced and tied at another point. Catheters were made by joining a PE-10 tube (pulled on 1 end) to a PE-50 tube. All catheters were filled with sterile saline before surgery.

Once the catheter was tied in place, lidocaine was administered along the skin incision. Next, ISO was reduced to 1% for 5 min. It was important that the animal was not touched or moved during this time. An arterial blood sample was taken at the 5-min point (*n* = 10 CD-1, *n* = 8 C57BL/6J) followed by a return to 2% ISO. In a separate series of experiments, C57BL/6J mice (*n* = 5) were tested at 11 min of 1% ISO followed by a return to 2% ISO. This 11-min sample was chosen based on the breathing frequency response observed in mice by the 11th min (Fig. 3). A plug was inserted into the end of the catheter, and ISO was stopped. Mice were allowed to recover in their cage for 60–90 min following the surgery before a second arterial blood sample was taken in CD-1 mice. In the C57BL/6J mice, NO ISO samples were taken from one cohort of mice, and 1% ISO samples were taken from another cohort of mice (individual mice did not have multiple samples). All C57BL/6J mice had been involved in previous behavioral experiments (barometric plethysmography, blood pressure, and/or pulse oximetry). NO ISO samples were only initiated if mice were eating, ambulating, and appeared overall to be recovered from the anesthesia. Extreme care was taken to confirm that mice were not agitated and were, in fact, resting calmly in their cages. The C57BL/6J mice were more prone to excitement, easily disturbed, and often pulled at their catheter, sometimes with success (immediately ending the experiment). At the time of collection, all blood samples (0.10–0.13 ml) were placed into a 1-ml syringe and then into a CG8+ i-STAT cartridge (Abbott, Abbott Park, IL) for measuring pH and $Pa_{CO}{}_{{}_{2}}$. After the arterial sample, mice were placed back under ISO anesthesia, the catheter was removed, and the artery was tied. The incision was sutured closed, Rimadyl was administered (10 mg/kg sc), and mice were allowed to recover in their home cage.

All mice recovered well with this intervention, and most walked normally following the surgery. A small number did pick at the foot fed by the tested femoral artery and had an altered gait but ate and drank normally with an otherwise typical appearance.

### Pulse Oximetry

C57BL/6J mice (*n* = 7) were tested with the STARR Life Sciences MouseOx Plus system (Oakmont, PA). Fur was removed around the neck to allow the pulse oximetry collar to obtain readings. Mice were induced at 3% ISO for 3 min in the induction chamber and then moved to the surgical setup where they received 2% ISO for 27 min through a nose cone. This timeframe was used as it was the typical amount of time to the first 1% ISO sample in arterial blood sampling experiments. Next, mice were administered 1% ISO for 5 min, then the ISO was shut off and mice were moved to a circular chamber (similar in size to the home cage) with food, water, and bedding, and post-ISO data were collected for 3–4 h. Pulse oximetry data were collected during the 2% ISO exposure to the end of the experiment. These experiments were conducted for two reasons: *1*) to follow oxygen saturation during NO ISO to determine an optimal time for the awake sample and *2*) to identify any drops in oxygen saturation while the mouse was on the surgical table.

### Piezoelectric

Breathing frequency was measured using a Piezoelectric Disk (Vktech, Amazon.com, Seattle, WA) to monitor changes in mechanical force. The disk was connected to an iWorx signal conditioner/amplifier (Dover, NH), and iWorx LabScribe computer software (version 3) was used to record and analyze the data. C57BL/6J mice (*n* = 6) were induced at 3% ISO for 3 min in the induction chamber and then moved to the surgical setup, at which point they received 2% ISO (flow ~600 ml/min) for 27 min through a nose cone with a vacuum of ~100 ml/min. These conditions were the same as those used for implantation of the arterial femoral catheter. Next, mice were administered 1% ISO for 15 min to observe any possible changes in breathing frequency observed on the surgical table.

### Barometric Plethysmography

Unrestrained breathing experiments were performed to confirm previous results (27, 28) using our vaporizer, vacuum, and gas mixer setup. We were also interested in the response to hypoxia with 1% ISO, which had not previously been reported. Experiments were performed during the first hours of the light cycle, with two C57BL/6J (*n* = 8 total) mice tested at a time. Buxco mouse chambers (Data Sciences International, DSI; New Brighton, MN) were used with the reference chamber and animal chamber attached via SMC soft polyurethane tubing (model TU0604BU-20; Yorba Linda, CA) to the ports of a Validyne transducer (DP45; Northridge, CA) to monitor pressure changes. The Validyne voltage output was signal-conditioned through an ACQ-7700 (DSI) and further analyzed with Ponemah software (DSI) using the Drorbaugh and Fenn equation for tidal volume (12). The barometric plethysmography system was calibrated before each use with a two-point calibration. The span on the software was then set to allow for a maximum (mouse) peak inspiratory flow added to the calibrated gas flow. With this method, we had a strong resolution while avoiding any possible clipping of data. Flow for each chamber was held at ~300 ml/min using a mixture of nitrogen, oxygen, and carbon dioxide from pressurized canisters (Purity Plus Specialty Gases Grade 5.0 ultra pure) that were mixed with a three-channel gas blender (GB 103; MCQ Instruments, Rome, Italy). Flow was measured before entering the bias flow opening on the mouse chambers using TSI 4100 Series Flowmeters (Shoreview, MN) that had been factory-calibrated within 1 yr. Each chamber was equipped with a humidity and temperature probe that was incorporated into the Ponemah software for calculation of tidal volume and minute ventilation. A barometric pressure probe was also used for monitoring and was connected to the ACQ-7700 signal conditioner and Ponemah software. In general, we observed very few fluctuations in barometric pressure with this setup, but this probe offered an additional confirmation. Humidity and temperature of the room were also recorded (Traceable; Thermo Fisher Scientific, Waltham, MA) to confirm accuracy of the chamber probes and to monitor any swings in chamber vs. room temperature. Following data collection, breaths were analyzed offline using Ponemah (analysis software service pack versions 4.0 and 5.0) review mode to quantify breathing frequency, tidal volume, minute ventilation, and the ratio of tidal volume to inspiratory time. Mice were tested during *hours 2*–*5* of the light cycle for consistency in circadian rhythms (16, 30).

#### Conscious protocol.

NO ISO plethysmography trials were conducted in male C57BL/6J mice. After calibration of the system, mice were placed into the *barometric plethysmography* chamber receiving ~300 ml/min pressurized air (20.93% O_2_-balance N_2_; mixed with MCQ Instruments GB 103) with a vacuum of ~100 ml/min from the chamber. Baseline was identified as 5 min of quiet breathing when mice were breathing air and appeared awake, calm, and conscious with no grooming or sniffing (had minimal movement within the chamber, but eyes were open and appeared to be awake). This typically took 90 min to achieve but ranged from ~40 to 100 min. After baseline with air, mice were exposed to a hypoxic challenge (10% O_2_-balance N_2_) for 5 min, then a 5-min recovery period with air, followed by 5 min of hypercapnic gas (5% CO_2_-balance air). A 5-min recovery period with air was then collected before mice were removed from the chamber.

#### Anesthetized protocol.

ISO barometric plethysmography trials were conducted 1 wk before NO ISO barometric plethysmography trials. ISO (NDC 11695-6776-2; Henry Schein, Melville, NY) was vaporized, and the apparatus was kept under the hood (Highland Medical Equipment, Temecula, CA). Preblended gases (GB 103; MCQ Instruments) were fed through the input of the ISO vaporizer using SMC soft polyurethane tubing (model TUS0604Y-20), and the output tubing went through a TSI Flowmeter and into the mouse chambers. A constant vacuum was connected to each plethysmography chamber to combat the possibility that the ISO anesthesia was settling and collecting in the bottom of the chamber. It was left on for the duration of data collection to maximize consistency across trials.

Calibrations and gas flows were identical in the anesthetized and conscious protocols. However, 1 h before beginning each anesthesia experiment, a heating pad on the medium setting was placed under the plethysmography chamber and the internal Plexiglas grates were removed. These modifications to the protocol were used to keep the mouse’s body temperature in a normal range, as anesthesia is known to reduce body temperature (33). Calibration in the anesthetized trials was done after removal of the grates; the maximum flow used for calibration was not impacted by this procedure. In a subset of mice, body temperature was recorded using the LifeChip system (Destron Fearing, Airport, TX) every 10 min during baseline and each minute during ISO exposure. Body temperature was maintained within 37 (0.46)°C. Ambient temperature inside the chamber was measured and remained consistent across trials.

The mice were placed inside and acclimated to the chamber for 30 min with air, then ISO induction began for 5 min at 2% ISO (in air), followed by 5 min at 1% ISO (in air). While still receiving 1% ISO, mice were exposed to a hypoxic challenge (10% O_2_-balance N_2_) for 5 min, then 5 min of air followed by 5 min of hypercapnic gas (5% CO_2_-balance air) and 5 min of air. Finally, the mice were exposed to air with no ISO for 5 min. Mice were then removed from the chamber.

#### Data analysis.

The last minute of each gas exposure was analyzed using Ponemah analysis software. It is not recommended to run anesthetics through the metabolic analyzers, so we did not collect oxygen consumption or CO_2_ consumption. However, a trial was run without anesthesia, using AEI metabolic analyzers (Pittsburgh, PA), to ensure that the previous gas concentration was completely flushed out of the chambers and that the mice were being exposed to the new gas mixture in a timely manner (maximum time to equilibrium was 2 min 25 s, average time to equilibrium was 1 min 57 s). Therefore, we are confident that the last minute was most representative of the described gas concentration (air, hypoxic gas, hypercapnic gas). Parameters measured during the last minute of each gas exposure included frequency, tidal volume, tidal volume/inspiratory time, and minute ventilation.

### Blood Pressure

C57BL/6J mice (*n* = 9) were brought to the animal behavior testing area 1 h before beginning trials to allow the mice to acclimate. Blood pressure was measured with the Kent Scientific Coda Blood Pressure System (Torrington, CT). The occlusion cuff of the volume-pressure recording system was placed at the base of the tail, and the volume-pressure recording cuff was placed below the occlusion cuff. Mice were placed in the restraint tube 5 min before data collection; the cuffs then began the inflation/deflation cycles. Each minute throughout the experiment, body temperature was monitored and maintained using the LifeChip temperature monitoring system and a heating pad.

Anesthetized trials were similar to the conscious trials but with the addition of 2 and 1% ISO. After cuff placement and acclimation, the mice were administered 2% ISO for 5 min using a nose cone to direct the anesthesia into the chamber. Then, the ISO was turned down to 1%. After 2 min, the blood pressure measurements began.

A trial was considered successful if ≥7 of the 15 cycles of inflation/deflation cycles were accepted by the software. The mean values from the 7–15 cycles were calculated for each mouse (diastolic blood pressure, systolic blood pressure, and mean arterial pressure). All mice were tested once with ISO.

### Statistics

To identify possible pattern of breathing differences between NO ISO and ISO mice, a repeated-measures two-way analysis of variance (ANOVA) was used with anesthetic as the group and gas exposure as the repeated-measures factor. This analysis was selected to account for the fact that the same mice were measured under each of the gas exposures, with both NO ISO and ISO conditions. Bonferroni-corrected post hoc tests using a paired *t*-test were calculated to determine specific differences between the gas exposures of interest. A Student’s *t*-test identified possible differences between pH and $Pa_{CO}{}_{{}_{2}}$ in the CD-1 mice and blood pressure, arterial oxygen saturation, heart rate, weight, pH, and $Pa_{CO}{}_{{}_{2}}$ between NO ISO and 5-min 1% ISO in C57BL/6J mice. Paired *t*-tests were used when comparisons were across observations of the same mice, and significance levels were Bonferroni-corrected when multiple comparisons were made.

Levene’s test was used to identify differences in standard deviations for oxygen saturation for each mouse individually, and an *F* test was run to compare standard deviations for oxygen saturation between the two conditions overall. In a later series of experiments, we tested C57BL/6J mice following 11 min of ISO. A one-way ANOVA was used to identify differences between arterial $Pa_{CO}{}_{{}_{2}}$ and pH across blood samples (NO ISO, 5-min ISO, and 11-min ISO) and to compare arterial blood oxygen saturation across exposures (2% ISO, 1% ISO, and NO ISO) in C57BL/6J mice. Significance was selected a priori to be *P* < 0.05. R and SPSS software were used to perform statistical analyses. Data are reported as means (SD); all error bars in the tables and graphs represent standard deviation.

## RESULTS

### Blood Sampling

Blood sampling in CD-1 mice uncovered no difference in $Pa_{CO}{}_{{}_{2}}$ or pH between NO ISO and mice administered 5 min of 1% ISO (Table 1). The same CD-1 mice had samples collected with NO ISO and ISO. NO ISO and 5-min 1% ISO arterial blood sampling in C57BL/6J mice (unique mice in each group) showed differences with $Pa_{CO}{}_{{}_{2}}$ [NO ISO: 38.78 (1.86), 5-min 1% ISO: 35.84 (1.89); *P* = 0.019] and pH [NO ISO: 7.43 (0.02), 5-min 1% ISO: 7.38 (0.03); *P* = 0.007], although the values remained in the typical physiological range of health (pH 7.2–7.4; Ref. 18). We added an experiment with C57BL/6J mice (*n* = 5) with samples taken during the 11th min of 1% ISO exposure. There were no differences in pH (*P* = 0.97) or $Pa_{CO}{}_{{}_{2}}$ (*P* = 0.96) among the NO ISO, 5-min 1% ISO, and 11-min 1% ISO samples.

**Table 1.** Conscious and 1% isoflurane blood sampling data

	Arterial Blood			
	C57BL/6J		CD-1	
	pH	$Pa_{CO}{}_{{}_{2}}$, mmHg	pH	$Pa_{CO}{}_{{}_{2}}$, mmHg
NO ISO	7.43 (0.02)	38.8 (1.9)	7.43 (0.02)	36.7 (2.4)
5-min 1% ISO	7.38 (0.03)	35.2 (2.3)	7.43 (0.02)	34.8 (2.6)
11-min 1% ISO	7.39 (0.06)	38.2 (9.7)		
*P* Values	0.97	0.96	0.44	0.12

Data are represented by means (SD). pH and arterial Pco_2_ ($Pa_{CO}{}_{{}_{2}}$) were not different between conscious (NO ISO) and 1% isoflurane (1% ISO) conditions in CD-1 and C57BL/6J mice. Arterial blood gas data were measured for CD-1 (NO ISO: *n* = 10, 1% ISO: *n* = 9) and C57BL/6J mice (NO ISO: *n* = 5, 5-min 1% ISO: *n* = 12, 11-min 1% ISO: *n* = 7). No significant differences emerged using an ANOVA for C57BL/6J or Student’s *t*-test for CD-1.

### Pulse Oximetry

**Fig. 2.**
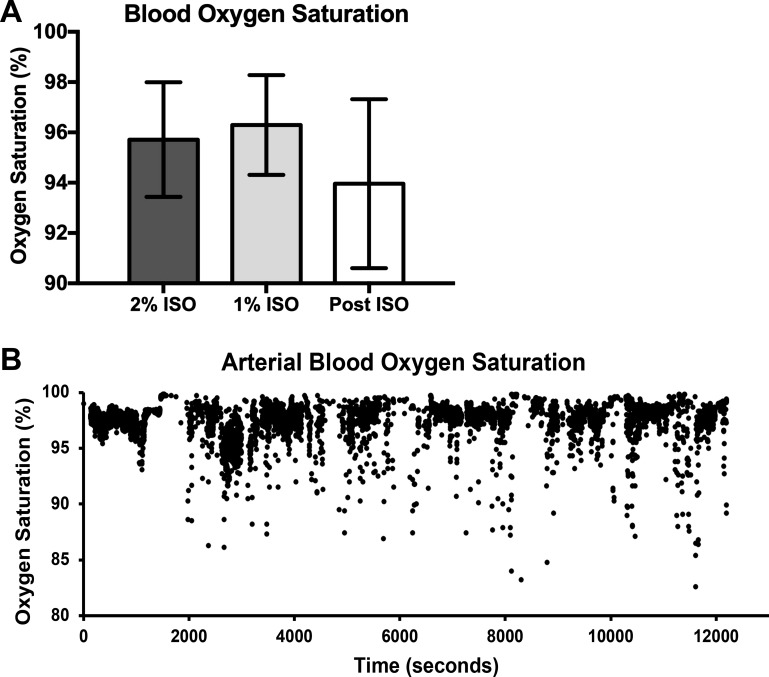
Arterial blood oxygen saturation in C57BL/6J mice was not different among conscious (NO ISO), 2% isoflurane (2% ISO), and 1% isoflurane (1% ISO) exposures. *A*: mean pulse oximetry data for C57BL/6J mice (*n* = 7). Mice were exposed to 3% isoflurane (3% ISO) for induction of anesthesia, then 2% ISO for 27 min followed by 5 min of 1% ISO and monitored for ≤4 h during recovery with air. Five-minute averages of 2 and 1% ISO are represented. After isoflurane (Post ISO) is a 1-h average, using *minutes 90*–*120* from the start of the experiment (60 min of ISO “washout” were allowed before this average). There were no significant differences among 2% ISO, 1% ISO, and Post ISO (1-way ANOVA, *P* = 0.64). Variance was not different between 1% ISO (5 min) and Post ISO (5 min) arterial blood oxygen saturation (*F* test, *P* = 0.18). Five of seven mice showed higher variation with NO ISO (4 h) vs. 1% ISO when comparing individual responses using Levene’s test. Data are represented by means ± SD. *B*: continuous pulse oximetry data for a single C57BL/6J mouse over a 12,000-s span (200 min). On the tracing, the mouse was exposed to 2% ISO from 0 to 1,095 s and 1% ISO from 1,096 to 1,395 s. At this time, the ISO was shut off and the mouse was exposed to room air for the remainder of the 12,000 s.

Pulse oximetry was used to collect arterial oxygen saturation over time on a minute-to-minute basis to monitor the dynamic changes of oxygen levels in the blood. C57BL/6J mice had similar oxygen saturation when exposed to 2% ISO, 1% ISO, and NO ISO in air (*P* = 0.64; Fig. 2*A*). When analyzed individually, five of seven mice showed higher variation with NO ISO vs. 1% ISO when comparing with Levene’s test. However, when using the *F* test, no difference in the standard deviations was observed between groups. It should be noted that the *F* test used only 5 min of NO ISO to compare directly with the 5 min of 1% ISO. Levene’s test allowed us to include all 4 h of data collection with NO ISO. A tracing from a representative mouse is shown in Fig. 2*B*.

### Breathing Frequency Using a Nose Cone

**Fig. 3.**
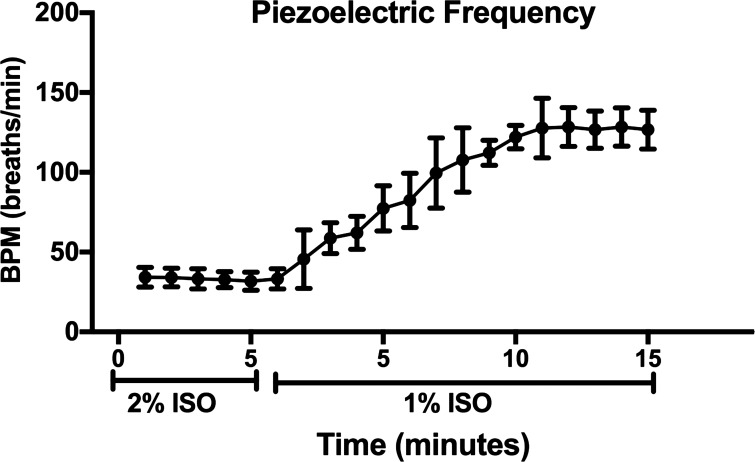
Breathing frequency for C57BL/6J mice over the course of 2% isoflurane (2% ISO) and a longer duration of 1% isoflurane (1% ISO) exposure displays a plateau in frequency at approximately 10–11 min of 1% ISO. Breathing frequency (breaths/min; BPM) was measured using a Piezoelectric Disk with the surgical setup for C57BL/6J mice. Mice were administered 3 min of 3% ISO for induction and then 27 min of 2% ISO in air through the nose cone followed by 1% ISO. The last 5 min of 2% ISO (*n* = 6) were averaged, and the duration of 1% ISO was analyzed and averaged for each minute. One animal was only exposed to 10 min of 1% ISO, so a sample size of 5 is represented for *minutes 16*–*20* during 1% ISO. Data are represented by means ± SD.

Frequency was quantified using a Piezoelectric Disk, and data were averaged for the last 5 min of 2% (30-min total exposure of deeper anesthesia was administered) and each minute of 1% ISO (15-min exposure). Under 2% ISO, C57BL/6J mice had a depressed breathing frequency that rose continuously when administered 1% ISO until it plateaued at 132 (39) breaths/min during *minutes 10*–*15* of 1% exposure (Fig. 3).

### Breathing Patterns

**Fig. 4.**
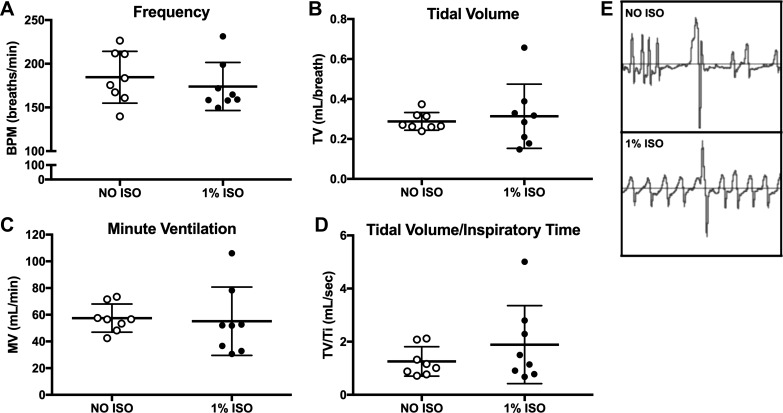
Breathing frequency, tidal volume, minute ventilation, and tidal volume/inspiratory time were not different in conscious (NO ISO) and 1% isoflurane (1% ISO) C57BL/6J mice breathing air. Baseline NO ISO and 1% ISO breathing measures for C57BL/6J mice (*n* = 8/group) are shown. Breathing frequency (*A*; breaths/min; BPM), tidal volume (*B*; TV; ml/breath), minute ventilation (*C*; MV; ml/min), and tidal volume/inspiratory time (*D*; TV/T_i_; ml/s) are shown for NO ISO and 1% ISO mice breathing air. No statistically significant differences in frequency (*P* = 0.47), TV (*P* = 0.67), MV (*P* = 0.83), or TV/T_i_ (*P* = 0.69) were found between groups using Student’s *t*-tests. Data are represented as means ± SD. *E*: a representative tracing of NO ISO and 1% ISO augmented breaths for a single C57BL/6J mouse. Windows depict the same timeframe for both. Augmented breaths were quantified for each mouse for the 5-min NO ISO baseline and the 5-min 1% ISO exposure. These values were not different (*P* = 0.33) when using a Student’s *t*-test, although the typical postsigh apnea was not present in 1% ISO.

In the C57BL/6J mice, quiet breathing with air in the NO ISO and 1% ISO exposures was not different for frequency (*P* = 0.50; Fig. 4*A*), tidal volume (*P* = 0.69; Fig. 4*B*), or minute ventilation (*P* = 0.83; Fig. 4*C*). Body weights were not different [NO ISO: 27.8 (1.8) g, 1% ISO: 27.3 (2.1) g; *P* = 0.59] between anesthetized and conscious trials, so we did not normalize for body weight. Tidal volume/inspiratory time, a measure of the neural drive to breathe (9), was not different when comparing NO ISO and 1% ISO in mice breathing air (Fig. 4*D*; *P* = 0.33). Based on these experiments, breathing air with NO ISO or 1% ISO does not change the pattern of breathing in C57BL/6J mice. These findings regarding air breathing are in line with previous reports (27, 28).

Augmented breaths, commonly known as sighs, are a respiratory reflex observed in mice (40) and other organisms. NO ISO augmented breaths have the characteristic appearance of apnea before and after a very large breath (Fig. 4*E*). In the ISO-anesthetized mice, the augmented breaths did not exhibit the classic characteristic of apnea before and after the breath (Fig. 4*E*), but they were identifiable by the larger size. The frequency of augmented breaths in the 5 min of NO ISO quiet breathing [0.28 (0.39) augmented breaths/min] was not different compared with 5 min of 1% ISO exposure [0.29 (0.66) augmented breaths/min; *P* = 0.99].

**Fig. 5.**
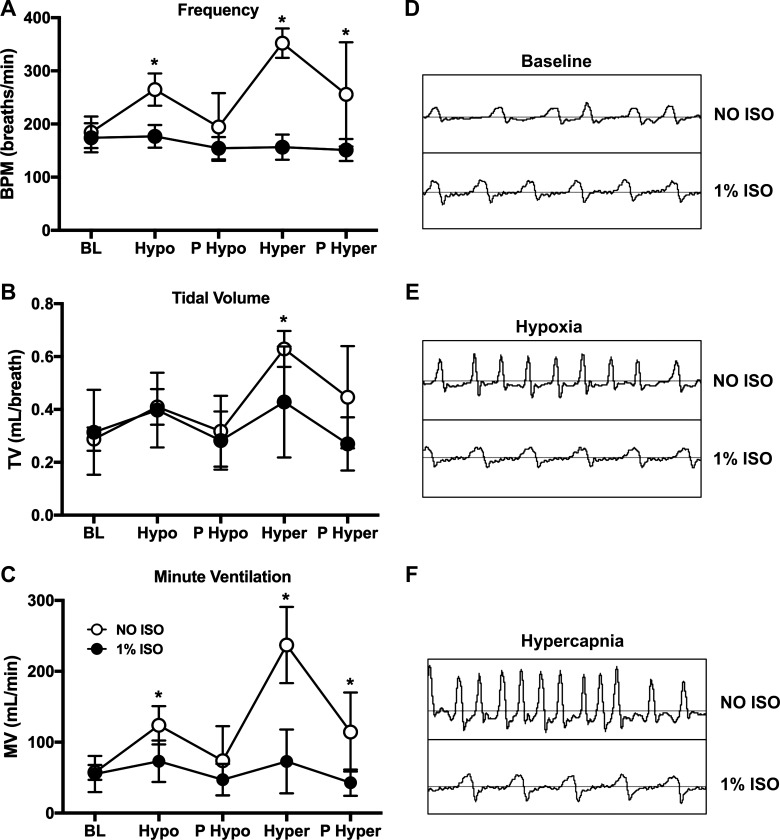
Breathing frequency, tidal volume, and minute ventilation were not different in conscious (NO ISO) and 1% isoflurane (1% ISO) C57BL/6J mice exposed to air, but the pattern of breathing was different between NO ISO and 1% ISO when mice were exposed to hypoxia and hypercapnia. NO ISO and 1% ISO breathing measures across gas exposures. Breathing frequency (*A*; breaths/min; BPM), tidal volume (*B*; TV; ml/breath), and minute ventilation (*C*; MV; ml/min) for gas exposures administered to C57BL/6J mice (*n* = 8 total) are shown. Final minute of each gas exposure is represented on the graph except for baseline (BL), which is a 5-min average. Representative breathing trace from a single mouse is shown for baseline (*D*), hypoxia (*E*), and hypercapnia (*F*); all scales are the same. Data were analyzed using repeated-measures analysis of variance (ANOVA; BPM, *P* < 0.001; TV, *P* = 0.004; MV, *P* < 0.001). *P < 0.05, NO ISO vs. 1% ISO. Hyper, hypercapnia = 5% CO_2_-balance air; Hypo, hypoxia = 10% O_2_-balance N_2_; P Hyper, posthypercapnia recovery period (air); P Hypo, posthypoxia recovery period (air). Data are represented as means ± SD.

The upper respiratory capacity was tested by exposing mice to the known respiratory stressors of hypoxic and hypercapnic gas (Fig. 5). A blunted hypoxic response was found with ISO; this was due to a lower frequency (*P* < 0.001) and resulting minute ventilation (*P* = 0.006; Fig. 5, *A* and *C*). Hypercapnic gas also revealed a reduced response to ISO with frequency (*P* < 0.001), tidal volume (*P* = 0.03), and minute ventilation (*P* < 0.001) differences (Fig. 5, *A*–*C*).

### Blood Pressure and Heart Rate

Blood pressure was monitored to identify possible changes in the cardiovascular system, as these alterations could influence gas exchange (1). A decrease in systemic blood pressure is often associated with higher levels of ISO (41), although pulmonary pressures and flow were not different in an ISO study using dogs (25).

To determine potential changes in systemic cardiovascular function due to 1% ISO, tail blood pressure and heart rate were monitored in C57BL/6J mice with NO ISO and 1% ISO. Mean arterial blood pressure (*P* = 0.10), systolic blood pressure (*P* = 0.10), diastolic blood pressure (*P* = 0.10), and heart rate (*P* = 0.10) were not different in NO ISO and 1% ISO-anesthetized animals (Table 2).

**Table 2.** Blood pressure and heart rate in conscious and 1% isoflurane-exposed mice

	Mean Arterial BP, mmHg		Systolic BP, mmHg		Diastolic BP, mmHg		Heart Rate, beats/min	
	NO ISO	1% ISO	NO ISO	1% ISO	NO ISO	1% ISO	NO ISO	1% ISO
	86 (6)	81 (9)	102 (7)	97 (10)	78 (6)	74 (9)	475 (95)	526 (61)
*P* Values	0.10		0.10		0.10		0.10	

Data are represented by means (SD). Cardiovascular measurements were not different between conscious (NO ISO) and 1% isoflurane (1% ISO) conditions in C57BL/6J mice. C57BL/6J mice (*n* = 9/group) were tested for blood pressure (BP) and heart rate using volume-pressure recording tail cuff. No significant differences were observed between NO ISO and 1% ISO groups using Student’s *t*-test. Bonferroni-corrected significance level of 0.05/4 = 0.0125.

## DISCUSSION

This investigation studied the impact of 1% ISO on pH and $Pa_{CO}{}_{{}_{2}}$ in C57BL/6J and CD-1 male mice with additional breathing and cardiovascular measures in C57BL/6J mice. Blood sampling revealed no difference in pH and $Pa_{CO}{}_{{}_{2}}$ between NO ISO and 1% ISO CD-1 mice using the 5-min time point. Thus, when testing CD-1 mice, 5 min of 1% ISO may be a comparable alternative to NO ISO blood sampling due to the lack of observed differences. In C57BL/6J mice, additional experiments were required to find the optimal time point with 1% ISO that was comparable with awake samples.

Blood sampling during the 5th min of 1% ISO exposure in C57BL/6J mice did reveal lower pH and $Pa_{CO}{}_{{}_{2}}$ vs. NO ISO in a paired *t*-test, although these values remained within a normal range (18). In support of the typical physiological ranges, breathing patterns were not different in NO ISO vs. 5 min 1% ISO C57BL/6J mice breathing air. Additionally, blood pressure and heart rate were not different in NO ISO and 5 min of 1% ISO C57BL/6J mice exposed to air. In another series of blood sampling in C57BL/6J mice at 11 min of 1% ISO, we found that pH and $Pa_{CO}{}_{{}_{2}}$ were similar to NO ISO and 5 min of 1% ISO-exposed mice. Therefore, this time point for arterial blood sampling may be preferred when testing C57BL/6J mice breathing air.

When mice were administered hypoxic and hypercapnic gas, minute ventilation and frequency were lower in 1% ISO compared with NO ISO C57BL/6J mice. For these reasons, 1% ISO blood sampling would not be recommended for mice exposed to respiratory stressors. The lower breathing frequency in the 1% ISO group exposed to hypoxic and hypercapnic gas suggests a depression of the neural drive to breathe or changes in chemosensation (or both) at O_2_ and CO_2_ exposures outside the range of normal air. The quantity of augmented breaths was the same between NO ISO and 1% ISO C57BL/6J mice. However, in line with a neural component, the shape of the augmented breaths with 1% ISO was unique. Augmented breaths are under neural control (26), so the difference in the shape of NO ISO and ISO-anesthetized sighs may involve modifications in the anesthetized nervous system.

In a series of experiments, Massey et al. (27) investigated the impact of 1% ISO on chemosensitivity. Although their study used 50% O_2_ and our protocol administered normoxic conditions during hypercapnic exposure, we came to similar conclusions that the 1% ISO-anesthetized mice exhibited a blunted ventilatory response to hypercapnic gas but similar pattern of breathing responses with room air. Massey et al. (27) found ISO abolished pH and $Pa_{CO}{}_{{}_{2}}$ chemosensitivity with hypercapnic exposure, which is in line with our findings of a blunted response to hypercapnic gas with 1% ISO. They attributed this change to a decrease in serotonin neuron firing frequency (27). With a decreased rate of firing in response to the increased CO_2_ concentration, the motor output resulting in breathing frequency likely would decrease as well.

Initial testing indicated a lower pH and $Pa_{CO}{}_{{}_{2}}$ in the 5-min blood sample in 1% ISO-anesthetized C57BL/6J mice. Of importance, these values were still in a typical window of overall health and did not indicate acidosis. To maintain homeostasis within the narrow physiological range of pH 7.2–7.4, breathing frequency will increase to expel more CO_2_ and pH will increase as blood becomes more basic. Because blood sampling provides an instantaneous snapshot of blood pH and $Pa_{CO}{}_{{}_{2}}$, we considered it possible the sample was taken at the transition point from 2 to 1% ISO, where 5 min was too short for 1% ISO sampling in C57BL/6J mice and a later time point may have been more appropriate (approximately 10–15 min). The CD-1 protocol, which resulted in similar arterial blood values in NO ISO and 1% ISO, was initially used in the C57BL/6J mice. However, C57BL/6J mice have lower metabolic rates than CD-1 mice (43), which could have caused the ISO to take longer to metabolize and flush out of the (C57BL/6J) mouse’s system. With a lower metabolic rate in C57BL/6J mice, we hypothesized the time point of the blood sample in CD-1 mice (5 min) may not be adequate in C57BL/6J mice. Further testing with a Piezoelectric Disk in C57BL/6J mice showed that by 11 min breathing frequency had plateaued at 132 (39) breaths/min. Therefore, a blood sample taken at this time point would be most representative of a conscious sample in the C57BL/6J mice. We conducted another series of blood sampling at the 11-min time point with 1% ISO in C57BL/6J mice; these data indicate similar blood chemistry to the NO ISO and 5-min 1% ISO sample.

Although we followed the 5-min 1% ISO blood sampling with an additional 11-min 1% ISO time point, we also investigated the time point of our NO ISO blood samples. To evaluate whether the conscious blood sample was taken too quickly following recovery in the C57BL/6J mice, oxygen saturation was measured in C57BL/6J mice to determine whether there was an ideal window of time for a conscious blood sample to be collected (i.e., immediately to 4 h postprocedure). Following catheter implantation, the mouse may have reduced breathing to slightly lower O_2_ levels and raise CO_2_. If oxygen saturation had not stabilized back to normal levels (95–98%) by 1 h of recovery, then it would be plausible the conscious blood sample was exhibiting slightly higher $Pa_{CO}{}_{{}_{2}}$ than the rest of the mice tested. However, we did not find this to be the case, and the 60- to 90-min time point appears to be more than adequate for a conscious sample in C57BL/6J mice. Our method for taking arterial samples in awake mice emphasized that mice were calm in their home cage. This meticulous protocol may explain the slightly higher $Pa_{CO}{}_{{}_{2}}$ we observed in awake mice compared with others in the literature (19, 20). However, our data are similar to those reported by Lee et al. (24), where mice were not disturbed during the sampling. Perhaps expected, the pulse oximetry experiments did show that arterial blood oxygen saturation tended to be more variable with NO ISO when comparing individual mice. In this case, Levene’s test was used during the NO ISO data collection compared with the 1% ISO exposure. This is likely due to increased activity of mice during NO ISO data collection.

Despite the fact that this research focused on CD-1 and C57BL/6J mice, the protocol can likely be adapted for use in other strains. One way researchers can utilize this method is by monitoring breathing frequency after the transition from 2 to 1% ISO. There seems to be a window between *minutes 5*–*10* after 2% ISO is stopped and during 1% ISO administration where the breathing frequency rapidly increases to a stable level (*minutes 10*–*12*) in C57BL/6J mice. By using a Piezoelectric Disk or other method to monitor breathing frequency, one can tailor this method of blood sampling to each individual mouse strain to ensure that the mice have adequately stabilized under 1% ISO to take a blood sample. We also suggest using this method with each specific mouse to help account for the variability of physiology.

### Historical Context of Arterial Blood Sampling

Arterial blood samples have been collected in a laboratory setting using human subjects and mammalian model organisms such as dogs, cats, and rabbits (2, 14, 17, 22). As research with rats became more prevalent, cannulation of different vessels was developed for arterial blood sampling in these rodents (31). With the emergence of mice as a common test subject, quantifying blood biochemistry became relevant and important for the field of respiratory neurobiology.

Arterial blood sampling in mice is anatomically similar to that for rats in that the carotid or femoral arteries are cannulated. However, behaviorally, rats tend to be more docile than mice and can even be trained to remain still during imaging procedures such as an fMRI (13). The smaller size of mice compared with rats, their accompanying lower blood volume, and more fragile nature present multiple technical issues for conscious arterial sampling (3, 5). Iversen et al. (20) collected blood samples in conscious mice using an indwelling catheter implanted in the carotid artery. Despite their success, the mice exhibited altered breathing patterns, likely indicating stress during the procedure, thus highlighting the difficulty of conscious samples in mice.

Although it is often an essential portion of experimental design for mouse studies to use arterial cannulation, and subsequent awake sampling, there are distinct difficulties associated with this type of blood collection in small rodents. In our hands, mice are more prone to pulling on the catheter and becoming excited around an experimenter compared with rats. Therefore, the 1% arterial blood sampling reduces the complication of external stimuli that influence breathing behaviors and eliminates animal interest in the surgical site and catheter as a factor impacting the feasibility of collecting samples.

### Shortcomings

This study investigated blood sampling in CD-1 and C57BL/6J mice, with more thorough testing of cardiovascular and pattern of breathing responses in C57BL/6J mice. Arterial blood sampling only provides information regarding a snapshot of time, leaving short- and long-term physiological responses largely unknown. Further cardiovascular and respiratory investigations in CD-1 mice would offer more information about the impacts of 1% ISO on this strain.

In addition, the lack of metabolic data (oxygen consumption or CO_2_ consumption) is an important limitation since a possible lower metabolic rate could influence the demand for breathing rate and depth. Reductions in body temperature are a primary influencer of lower metabolic rate with anesthetics (reviewed in Ref. 38); we did control for this possibility by maintaining core temperature throughout our experiments. However, changes in metabolic rate are still plausible, especially at higher levels of ISO when breathing rates are lower.

To this end, using arterial blood sampling as the final measure of blood pH and $Pa_{CO}{}_{{}_{2}}$ remains the best way to confirm adequate ventilation following barometric plethysmography studies. To reinforce the use of 1% ISO arterial blood sampling as a way to monitor ventilation, additional testing in female mice, mice in different age groups, knockout mice, and transgenic mice would provide information about how this protocol translates across different cohorts of mice.

### Conclusion

1% ISO blood sampling in air appears to be a suitable alternative to NO ISO blood sampling in CD-1 and C57BL/6J male mice. The lack of differences with pulse oximetry and blood pressure between NO ISO and 1% ISO exposures also suggests minimal changes to these physiological systems that can influence ventilation. Although the pattern of breathing is similar in NO ISO and 1% ISO mice breathing air, exposure to hypoxic and hypercapnic gas with 1% ISO resulted in reduced ventilatory responses compared with NO ISO. Therefore, the reaction to these other gas exposures at 1% ISO is not representative of a conscious animal. Still, 1% ISO blood sampling in air is similar to NO ISO in CD-1 and C57BL/6J mice depending on the time point used for 1% ISO. In other strains, this protocol should be evaluated on a case-by-case basis where breathing frequency of each mouse would be monitored to find the optimal time to collect a blood sample. The greater oxygen saturation variability observed in conscious C57BL/6J mice (Levene’s test) 0–4 h post-ISO is not surprising for awake, freely moving mice; thus it is plausible that 1% ISO would offer a more repeatable procedure for monitoring blood gas changes across groups of mice breathing air.

## GRANTS

This work was funded by National Institute of Child Health and Human Development Grants 1-R15-HD-076379 and 1-R15-HD-076379-A1S1 (each to L. R. DeRuisseau). Additional support was granted by the Le Moyne College McDevitt Center Undergraduate Research Fellowship in Natural Science (to A. M. Loeven) and the Le Moyne College Department of Biological Sciences (to L. R. DeRuisseau, C. N. Receno, and A. M. Loeven).

## DISCLOSURES

No conflicts of interest, financial or otherwise, are declared by the authors.

## AUTHOR CONTRIBUTIONS

A.M.L. and L.R.D. conceived and designed research; A.M.L., C.N.R., and L.R.D. performed experiments; A.M.L., C.N.R., C.M.C., and L.R.D. analyzed data; A.M.L., C.N.R., C.M.C., and L.R.D. interpreted results of experiments; A.M.L., C.N.R., and L.R.D. prepared figures; A.M.L., C.N.R., and L.R.D. drafted manuscript; A.M.L., C.N.R., C.M.C., and L.R.D. edited and revised manuscript; A.M.L., C.N.R., C.M.C., and L.R.D. approved final version of manuscript.
